# BMSC-Derived Exosomes Inhibit Dexamethasone-Induced Muscle Atrophy *via* the miR-486-5p/FoxO1 Axis

**DOI:** 10.3389/fendo.2021.681267

**Published:** 2021-10-01

**Authors:** Ziyi Li, Chang Liu, Shilun Li, Ting Li, Yukun Li, Na Wang, Xiaoxue Bao, Peng Xue, Sijing Liu

**Affiliations:** ^1^ Department of Endocrinology, The Third Hospital of Hebei Medical University, Shijiazhuang, China; ^2^ Key Orthopaedic Biomechanics Laboratory of Hebei Province, Orthopedic Research Institution of Hebei Province, Shijiazhuang, China; ^3^ Department of Joint Surgery, The Third Hospital of Hebei Medical University, Shijiazhuang, China; ^4^ Institute of Biomedical Engineering, Chinese Academy of Medical Sciences and Peking Union Medical College, Tianjin, China; ^5^ Editorial Department of Hebei Medical University, Hebei Medical University, Shijiazhuang, China

**Keywords:** muscle atrophy, bone marrow mesenchymal stem cell, exosomes, miR-486-5p, FoxO1

## Abstract

Sarcopenia, characterized by reduced muscle function as well as muscle mass, has been a public health problem with increasing prevalence. It might result from aging, injury, hormone imbalance and other catabolic conditions. Recently, exosomes were considered to regulate muscle regeneration and protein synthesis. In order to confirm the effect of BMSC-derived exosomes (BMSC-Exos) on muscle, dexamethasone-induced muscle atrophy was built both *in vitro* and *in vivo*. In the present research, BMSC-Exos attenuated the decrease of myotube diameter induced by dexamethasone, indicating that BMSC-Exos played a protective role in skeletal muscle atrophy. Further mechanism analysis exhibited that the content of miR-486-5p in C2C12 myotubes was up-regulated after treated with BMSC-Exos. Meanwhile, BMSC-Exos markedly downregulated the nuclear translocation of FoxO1, which plays an important role in muscle differentiation and atrophy. Importantly, the miR-486-5p inhibitor reversed the decreased expression of FoxO1 induced by BMSC-Exos. In animal experiments, BMSC-Exos inhibited dexamethasone-induced muscle atrophy, and miR-486-5p inhibitor reversed the protective effect of BMSC-Exos. These results indicating that BMSC-derived exosomes inhibit dexamethasone-induced muscle atrophy *via* miR486-5p/Foxo1 Axis.

## Introduction

Sarcopenia, with the character of loss of physical performance and muscle mass, leads to increased risk of disability and mortality (1). Muscle fiber loss accelerates after 40 years old, and nearly 30% of muscle mass will be lost by 80 years of age (2). Sarcopenia is expected to affect more than 200 million people worldwide in the next 40 years according to the European Working Group on Sarcopenia in Older People (2). Recent studies have found that sarcopenia often occurs simultaneously with osteoporosis, indicating that there should be a close relationship between bone and muscle (3). Importantly, the crosstalk between skeletal muscle and bone plays an important role during the process of aging (4).

Bone marrow mesenchymal stem cells (BMSCs) are considered as ideal seed cells for tissue engineering. BMSCs transplantation was demonstrated to contribute to skeletal muscle regeneration in rat. However, the protective role might not be produced by the myogenic differentiation of BMSCs, but by a paracrine effect of BMSCs on muscle regeneration (5). Recently, exosomes, the small phospholipid bilayer structures that containing proteins, RNA and other active substances, are considered to mediate the crosstalk between cells (6). Exosomes can exert the effects on target cells by delivering signal factors and activating downstream signaling cascades. Exosomes, which were released by different type of cells, undertake a vital role in promoting muscle health (7–10). It has been certified that exosomes derived from human skeletal myoblasts could up-regulate myogenesis of human adipose stem cells (11). MSC-derived exosomes had also been indicated to promote the myogenesis of C2C12 cells *in vitro* (12). However, the underlying mechanism of this beneficial effect remains elusive.

In this study, BMSC-Exos attenuated the decrease of myotube diameter induced by dexamethasone. Further mechanism analysis showed that the miR-486-5p/FoxO1 axis mediates this protective effect. Moreover, we demonstrated that BMSC-Exos alleviated dexamethasone-induced muscle atrophy *in vivo.*

## Materials and Methods

### Cell Culture

All animal experiments in this research were approved by the ethics committee of the Third Hospital of Hebei Medical University. BMSCs were separated from bone marrow of 4 Balb/c male mice which were obtained from the animal experimental center of Hebei Medical University. In short, mice were killed off and soaked in 75% alcohol for disinfection. Then, the femur was obtained by dissecting both lower limbs, and the bone marrow was flushed out with PBS. The primary BMSCs were blown into a single cell suspension and subsequently cultured in DMEM media containing 10% heat-inactivated fetal bovine serum. The skeletal muscle cell line, C2C12 myotubes, were obtained from Procell (Wuhan, China) and incubated in DMEM high glucose medium. C2C12 myotubes were cultured under myogenic induction (2% horse serum) for 6 days, followed by treatment with PBS (con group), BMSC-Exos (Exos group), 50 µM DEX (DEX group) or 50 µM DEX with BMSC-Exos (DEX+ Exos group) for a further 24 h. All cells were cultured in a stable environment of 37°C and 5% CO2.

### Isolation and Identification of Exosome

The MinuteTM efficient exosome precipitation reagent (Inent Biotechnologies Company, Beijing, China) was used to separate exosomes form the conditioned medium of BMSCs according to the instruction. Briefly, the BMSCs were cultured in serum-free medium for more than 15 hours after washed by PBS. Then, the culture supernatant was collected. Removing cells from the collected supernatant low-speed centrifugation (5min, 1000g) before incubating with exosome precipitant. Finally, centrifuging the samples for 1 h at 10,000g and removing the supernatant. The exosomes were collected and stored for further use. The concentration of exosomes was determined using a BCA protein assay kit (Servicebio, Wuhan, China). Ten μg/ml of BMSC-Exos was used to treat C2C12 cells.

The character of exosomes derived from BMSCs were further identified. TEM (Hitachi HT7700 TEM, Tokyo, Japan) were used to observe the morphology and diameter of exosomes. Briefly, exosomes samples were aspirated with pipette gun and placed in carbon membrane copper mesh for 5 minutes, then the excess liquid was aspirated with filter paper. The samples were subsequently stained with 2% phosphotungstic acid for 2 mins. Finally, the images of exosomes were collected by TEM. The specific surface proteins of exosomes, CD63 and CD81, were detected by western blotting assay.

### Exosome Uptake Assay

Firstly, exosomes were stained with PKH26 diluent for 5 minutes. Secondly, the excess dye was neutralized with 10% bovine serum albumin before washing the labeled exosomes in PBS for 60 minutes. Finally, C2C12 cells were treated with the labeled exosomes for 24h, stained with 4’,6‐diamindino‐2‐phenylindole and phalloidin.

### MiRNA Inhibitor and Mimics Transfection

miR‐486‐5p inhibitor and mimics was transfected into BMSCs by Lipofectamine 3000 (TermoFisher Scientific, USA) according to the protocol. miR-486-5p inhibitor and miR-486-5p mimics were separately mixed with Lipofectamine 3000 for 20 min and then cultured with BMSCs for 6 hours. Then, the transfected medium was renewed by complete culture medium. Cells were harvested for further analysis after 48 h post-transfection. The miR-486-5p inhibitor and miR-486-5p mimics were all purchased from ZHONGSHI TONGTRU (Tianjin, China).

### Real-Time RT-PCR Analysis

For the analysis of mRNA, total RNA was extracted through TRIzol^®^ reagent (TIANGEN, Beijing, China) before reversed-transcribed into cDNA *via* RevertAid™First Strand cDNA synthesis Kit (Thermo, Waltham, USA) based on the instructions. Then, RT-PCR analysis was performed according to TIANGEN SuperReal PreMix Plus’ protocols.

For the analysis of miRNA, microRNA assay kit was purchased from ZHONGSHI TONGTRU (Tianjin, China) and performed following the manufacturer’s introductions.

Primers used in the present research are compiled as follows:

GAPDH: 5’-GCAAGTTCAACGGCACA‐3’, 5’‐CGCCAGTAGACTCCACGAC‐3’; Atrogin-1: 5’- AAGGCTGTTGGAGCTGATAGCA -3’, 5’- CACCCACATGTTAATGTTGCCC -3’; MuRF1: 5’‐TGTCTCACGTGTGAGGTGCCTA‐3’; 5’- CACCAGCATGGAGATGCAGTTAC -3’; MyoG: 5’- GTAGTAGGCGGTGTCGTAGC -3’, 5’‐CCACGATGGACGTAAGGGAG‐3’.

### Western Blotting

The nuclear protein extraction kit (Beyotime, China) was used to get total protein of samples. Then, 20μg protein was separated *via* 12% SDS-PAGE and transferred to PVDF membranes before being blocked with 5% milk for 1 hour at room temperature. The PVDF membranes were stained at 4°C overnight with antibodies specific for FoxO1 (1:1000, Abcam, England), MuRF1(1:1000, Abcam, England), MyoG (1:1000, Abcam, England) and Lamin B1 (1:1000, Abcam, England). Finally, Blots were then stained with fluorescence secondary antibodies (1:20,000, Rockland, USA) and analyzed by the Odyssey Infrared Imaging System (Li-COR Biosciences).

### Animals and Treatment

A total of 20 male mice were randomly divided into four groups: control (CON), dexamethasone (DEX), dexamethasone + BMSC-Exosomes (DEX +inhibitor control-Exos), dexamethasone +miR-486-5p-inhibitor-Exos (DEX +miR-486-5p-inhibitor-Exos) after adaptive feeding. Dexamethasone (5mg/kg) dissolved in normal saline and was intraperitoneal injected once a day for 2 weeks until the end of experiment. BMSCs-Exo and miR-486-5p-inhibitor-Exos (100 μg exosomes resuspended in PBS) were injected into the muscle of limbs at multi-points every two days. Mice were maintained under suitable growth environment with free access to food and water.

### Measurements the Mass and Strength of Muscle

After 2 weeks of treatment, grip strength and running test were performed to evaluate the muscle force. For grip strength, mice were allowed to grip the T-bar using forelimbs. Then, the animals’ tails were pulled directly toward the tester. The pull force for each mouse should keep the same. Grip strength was calculated as force divided by the final body weight (N/g). For running test, total running time and distance were recorded. Body weight and muscle composition of each mouse was measured at the end of muscle function test.

### Immunostaining Analysis and the Measurement of Fiber Size

The mice were sacrificed after grip strength analysis. Muscle tissue was cut into thick serial sections for further staining. For muscle histology, the samples were stained with HE staining. The images were captured using an optical microscope.

Immunostaining of laminin for muscle tissue sections was performed after fixation and permeation. The images were captured and processed with a fluorescence microscope and analyzed using Image J software.

### Statistical Analysis

For the experiments *in vitro*, the data were obtained from at least 3 replicated tests, and for the experiments *in vivo*, there were 5 mice in each group. The data were described by mean ± standard deviation. Students t-tests and one-way ANOVAs with Tukey’s *post hoc* test were performed to compare the data between groups as appropriate. Statistically significant was measured by p < 0.05.

## Results

### Characterization of Exosomes Derived From BMSCs

**Figure 1:**
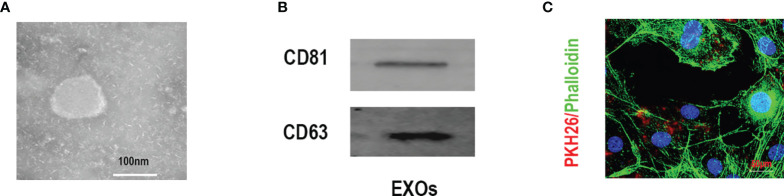
Characterization of exosomes derived from BMSCs. **(A)** Morphology identified by TEM, scale bar:100 nm. **(B)** The specific surface biomarkers of exosomes were analyzed by Western blotting assay. **(C)** The exosomes derived from BMSCs were marked with PKH26 and co-cultured with C2C12 myotubes, mbar = 50μm.

Typical spherical exosomes were captured by TEM (**Figure 1A**). The exosome-specific markers (CD63 and CD81) proteins were further detected by Western blotting analysis (**Figure 1B**). Subsequently, to further verify the endocytosis of BMSC-Exos in C2C12 myotubes, the fluorescent dye PKH26 was used to stain the BMSC-Exos. As shown in **Figure 1C**, PKH26-labeled BMSC-Exos were localized in the C2C12 region, which exhibiting the efficient internalization of the BMSC-Exos by C2C12 myotubes.

### BMSC-Exos Inhibited DEX-Induced Decreases in Myotube Diameter

**Figure 2:**
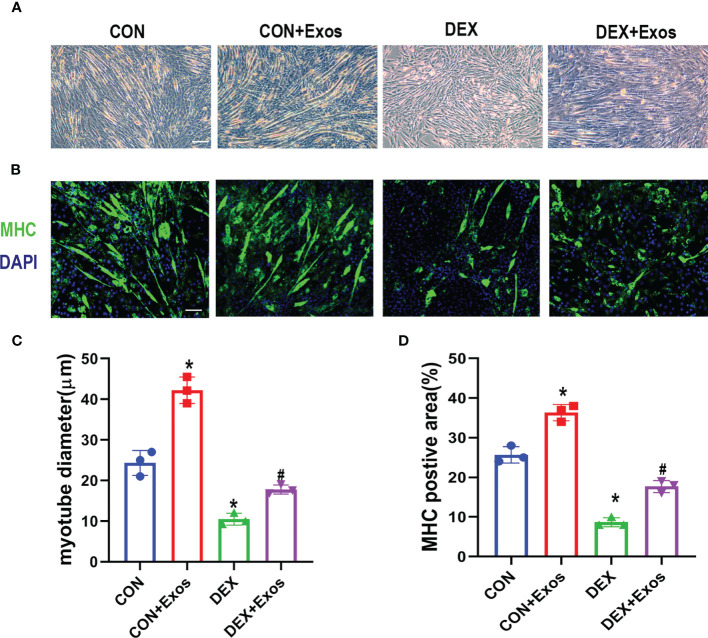
Exosomes derived from BMSCs inhibited DEX-induced myotube atrophy. **(A)** Representative images of C2C12 myotubes under the treatment of DEX and BMSC-Exos. Scale bar = 50µm **(B)** Immunofluorescence staining of Myosin high chain (MHC) in C2C12 cells with the treatment of DEX and BMSC-Exos. Scale bar = 50µm. **(C)** The myotube diameters among different groups were quantified (n = 3). **(D)** The MHC positive area among different groups were quantified (n = 3). *p < 0.05 compared to the control group, ^#^p < 0.05 compared to the DEX group.

Cell diameter were measured to confirm the positive role of BMSC-Exos on DEX-induced atrophy of C2C12 myotubes. C2C12 myotubes cultured under myogenic induction for 6 days, followed by treatment with PBS (con group), BMSC-Exos (Exos group), 50 µM DEX (DEX group) or 50 µM DEX with BMSC-Exos (DEX+ Exos group) for a further 24 h. The results showed that the cell diameter decreased due to the treatment of DEX, whereas the BMSC-Exos treatment attenuated the reduction of cell diameter by DEX (**Figures 2A, C**). Meanwhile, the results of MHC immunofluorescence staining indicated that BMSC-Exos significantly improve the quantity of myotubes (**Figures 2B, D**).

### BMSC-Exos Attenuated DEX-Induced FoxO1 Transmission Involved in Muscle Atrophy and Myogenesis

**Figure 3:**
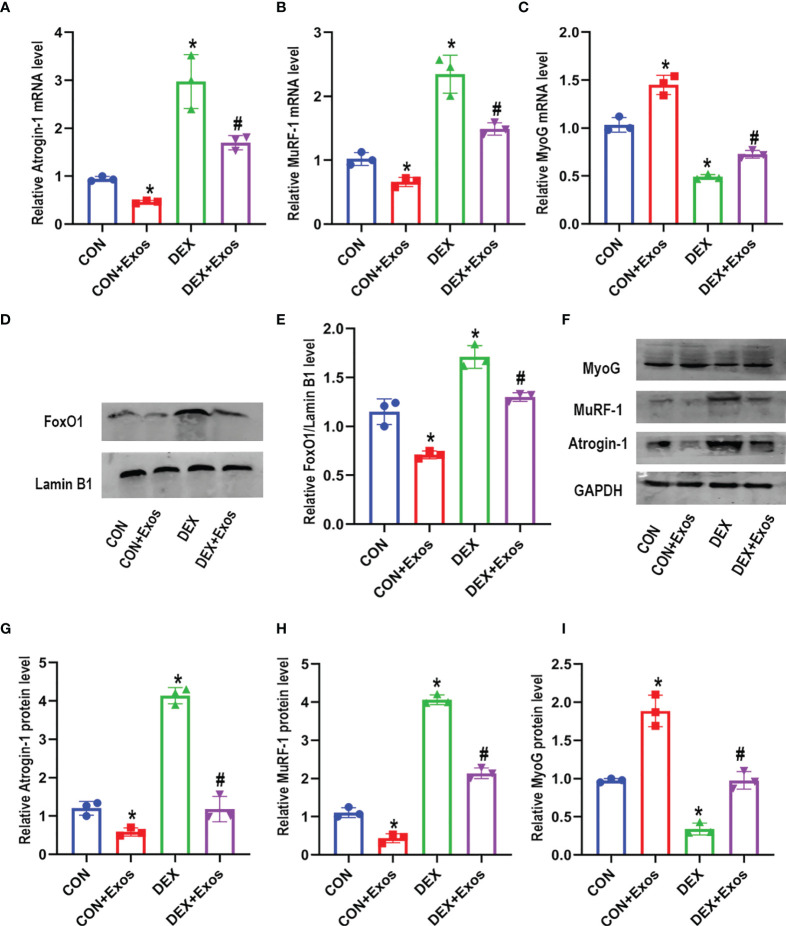
Exosomes derived from BMSCs regulated mRNA and protein expression involved in muscle atrophy and myogenesis. **(A)** Measurements of Atrogin-1 mRNA expression in different groups (n = 3). **(B)** Measurements of MuRF-1 mRNA expression in different groups (n = 3). **(C)** Measurements of MyoG mRNA expression in different groups (n = 3). **(D, E)** The protein level of FoxO1 in nuclear was measured by Western blot analysis (n = 3). Lamin B1 was used as the internal reference. **(F–I)** Measurements of Atrogin-1, MuRF1 and MyoG protein level in in different groups (n = 3). *p < 0.05 compared to the control group. ^#^p < 0.05 compared to the DEX group.

qRT-PCR analysis and western blot were performed to evaluate the effect of BMSC-Exos on muscle atrophy and myogenesis. The results demonstrated that DEX increased the level of atrogin-1 and MuRF1. Meanwhile, DEX administration induced down-regulation of myogenin (MyoG) (**Figures 3A**–**C**). However, the treatment of BMSC-Exos significantly reduced the up-regulation of mRNA and protein expression of atrogin-1 and MuRF1 induced by DEX. The decreased expression of DEX-evoked MyoG was also inhibited by BMSC-Exos (**Figures 3A**–**C**, **F**–**I**).

Western blotting analysis was performed after C2C12 myotubes were treated with BMSC-Exos to further verify the potential mechanisms. DEX treatment significantly increased the level of FoxO1 in nuclear compared to the control group. However, the increased expression of FoxO1 in nucleus was reversed by BMSC-Exos (**Figures 3D, E**). The above results indicated that BMSC-Exos could inhibit muscle atrophy induced by DEX *via* the regulation of FoxO1.

### MiR-486-5P Inhibitor Abolished the Effect of Exosomes Derived From BMSCs on Muscle Atrophy *In Vitro*

**Figure 4:**
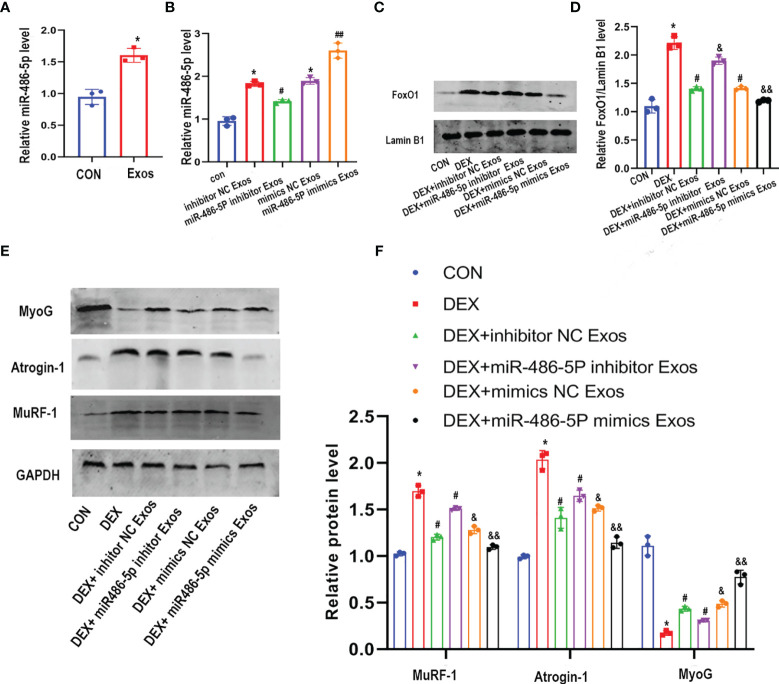
The inhibitor of miR-486-5P partially reversed the protective role of BMSC-Exos on DEX -induced myotube atrophy. **(A)** The level of miR-486-5p in C2C12 myotube after treated with con or con+BMSC-Exos were measured by qRT-PCR analysis (n=3). *p < 0.05 compared to the control group. **(B)** The expression of miR-486-5P in C2C12 myotubes after treated with different exos was measured by qRT-PCR analysis (n = 3). *p < 0.05 compared to the con group, ^#^ *p*< 0.05 compared to the inhibitor NC Exos group, ^##^ *p*< 0.05 compared to the mimics NC Exos group. **(C, D)** FoxO1 protein level in nuclear of C2C12 cells was measured and quantified (n=3). *p<0.05 compared to the con group, ^#^p < 0.05 compared to the DEX group, ^&^p < 0.05 compared to the DEX + inhibitor NC group, ^&&^p < 0.05 compared to the DEX+mimics NC group. **(E, F)** Measurements of Atrogin-1, MuRF1, and MyoG protein in DEX and Exos co-treated C2C12 myotube (n = 3). *p < 0.05 compared to the con group, ^#^p < 0.05 compared to the DEX group, ^&^p < 0.05 compared to the DEX + inhibitor NC group, ^&&^p < 0.05 compared to the DEX+mimics NC group.

In order to explore the mechanism that involved in the regulation of the BMSC-Exos on muscle atrophy, we then detected the expression of miR-486-5p. BMSC-Exos intervention led to an increased level of miR-486-5p in C2C12 myotubes (**Figure 4A**). To further investigate the role of miR-486-5p in BMSC-Exos-derived inhibition of muscle atrophy, inhibitor and mimics of miR-486-5p was transfected into BMSCs, respectively. miR-486-5p inhibitor Exos significantly inhibited the level of miR-486-5p in C2C12 myotubes compared to the inhibitor control Exos, while miR-486-5p mimics Exos significantly increased the level of miR-486-5p in C2C12 myotubes (**Figure 4B**). Furthermore, miR-486-5p inhibitor attenuated the BMSC-Exos induced decreases of FoxO1 expression and the subsequent changes of the expression of muscle atrophy markers (including Atrogin-1 and MuRF-1) as well as MyoG (**Figures 4C**–**F**). miR-486-5p mimics further up-regulated the protective effect of BMSC-Exos in muscle atrophy(**Figures 4C**–**F**). These results verified the protective role of BMSC-Exos on Dex-induced muscle atrophy was at least partly mediated by miR-486-5p.

### BMSC-Exos Increased Muscle Strength and Up-Regulated Muscle Weight *In Vivo*

**Figure 5:**
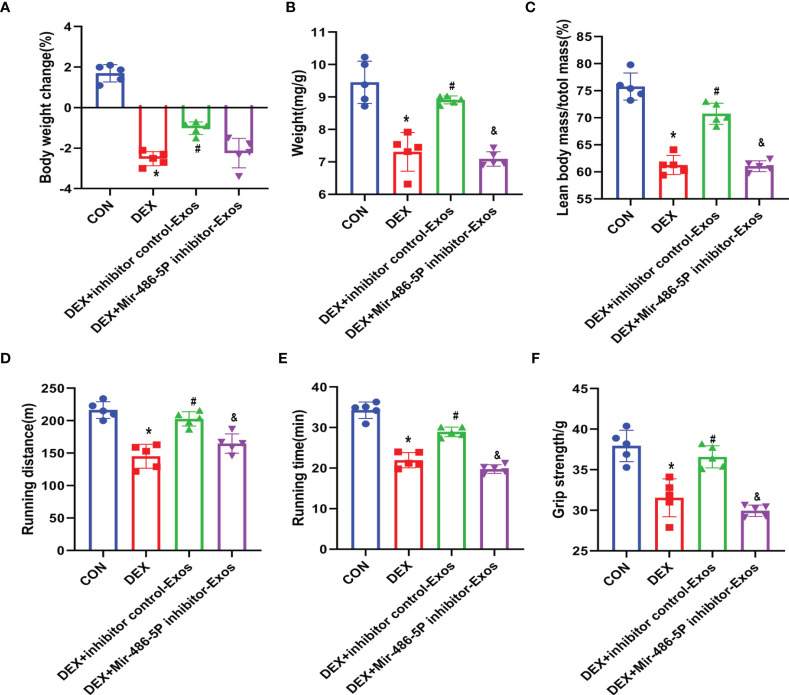
BMSC-Exos inhibited DEX-evoked decline in activity tolerance. **(A)** Body weight was measured after 14 days of treatment (n = 5). **(B)** Muscle mass comparison of different groups (n = 5). **(C)** The ratio of lean body mass to total mass (n = 5). **(D, E)** Running distance and active time in different groups (n = 5). **(F)** Grip strength was measured in different groups (n = 5). *p < 0.05 compared to the control group. ^#^p < 0.05 compared to the DEX group. ^&^p < 0.05 compared to the inhibitor NC group.

To further verify the effect of BMSC-Exos on DEX-evoked muscle atrophy, the mass as well as the function of muscle was measured. DEX treatment significantly decreased the animals’ body weight (**Figure 5A**), which could be reversed by inhibitor NC BMSC-Exos treatment. Meanwhile, the analysis of muscle weights indicated that BMSC-Exos significantly suppressed DEX-induced muscle atrophy (**Figure 5B**). The decreased ratio of lean body mass to total mass induced by DEX was partly reduced by the supplementation of inhibitor NC BMSC-Exos (**Figure 5C**). However, inhibiting miR486-5P in BMSC-Exos attenuated the protective role against DEX-induced muscle atrophy.

Muscle strength of the mice was measured by running test and grip strength (**Figures 5D**–**F**). DEX treatment decreased the activity time and muscle strength of mice, which was reversed by BMSC-Exos intervein. However, supplementation of miR-486-5p inhibitor-treated exosomes could not protect the DEX-induced muscle atrophy, and also could not increase the activity of DEX-treated mice.

### BMSC-Exos Prevents DEX-Evoked Muscle Atrophy *via* miR-486-5p *In Vivo*

**Figure 6:**
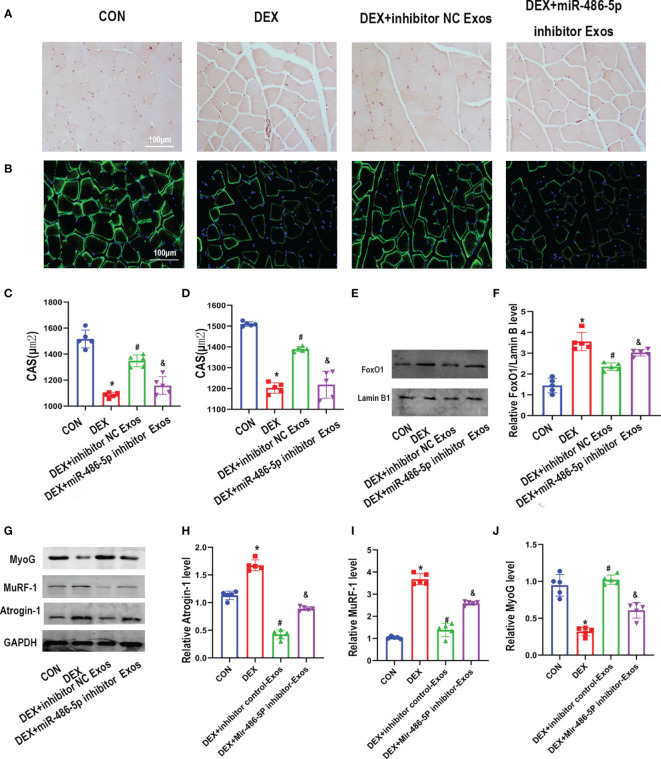
BMSC-Exos inhibited DEX-evoked muscle loss *in vivo*. **(A)** Cross-sectional area (CSA) of muscle were captured by H&E staining and quantified **(C)** (n = 5). scar bar = 100µm. **(B)** CSA of muscle were captured by immunostaining with laminin antibody and quantified **(D)** (n = 5). scar bar = 100µm. **(E, F)** The protein level of FoxO1 in nuclear was measured by Western blot analysis (n = 5). **(G–J)** The protein level of Atrogin-1,MuRF-1and MyoG in muscle was measured by Western blot analysis (n = 5). *p < 0.05 compared to the control group. ^#^p < 0.05 compared to the DEX group. ^&^p < 0.05 compared to the inhibitor NC group.

The histomorphological analysis indicated that DEX treatment decreased muscle fiber cross sectional area (CSA). BMSC-Exos treatment effectively increased CSA of muscle fiber. Meanwhile, supplementation of miR-486-5p inhibitor-treated exosomes decreased muscle fiber CSA compared with inhibitor control exosomes treatment (**Figures 6A**–**D**). Simultaneously, western blot analysis results revealed that inhibitor NC-treated BMSC-Exos other than miR-486-5p inhibitor-treated BMSC-Exos inhibited the DEX-evoked up-regulation of the nuclear FoxO1(**Figures 6E, F**). Meanwhile, the regulation of BMSC-Exos in Atrogin-1,MuRF-1and MyoG protein was also eliminated by miR-486-5p inhibitor (**Figures 6G–J**).

## Discussion

Skeletal muscle which accounts for more than 40% weight of the body, undertakes important tasks of physical strength, energy consumption and metabolism during life (13). However, muscle atrophy can be caused by drugs, diseases, injury as well as aging. Muscle loss results in decreased activity ability, increased fall risk and ultimately affect the quality of life. The reduction of muscle mass and function caused by muscle atrophy will increase morbidity and mortality. Moreover, as an important endocrine organ, muscle dysfunction can cause liver and fat metabolism disorder. Therefore, it is very important to prevent or delay the occurrence of muscle atrophy. However, there is no effective treatment for muscle atrophy (14). In the present research, exosomes secreted by BMSCs effectively ameliorates DEX-evoked muscle atrophy both *in vivo* and *vitro*. BMSC-Exos significantly downregulated the nuclear translocation of FoxO1, which plays a vital part in the regulation of skeletal muscle mass and myogenic differentiation. Furthermore, the protective effect of BMSC-Exos is due to the transfer of miR-486-5p from BMSC-Exos to muscle cells.

Dexamethasone, a common glucocorticoid drug, was used to treat various of inflammatory diseases (15). The effect of glucocorticoids in skeletal muscle is a double-edged sword (16). On the one side, glucocorticoids maintain the metabolic balance by coordinating the metabolism of glucose, lipid and protein. On the other hand, long-term as well as high doses usage of glucocorticoids might led to many passive effects, including muscular atrophy. The incidence rate of non-inflammatory myopathy is over 50% in patients who use steroid drugs for more than 4 weeks (17). Due to the multifactorial changes that contribute to the etiology of muscular atrophy, the underline pathogenesis of muscle atrophy is not completely understood. Current studies have found that DEX induced up-regulation of ROS, activation of the ubiquitin-proteasome and lysosomal pathways *via* the glucocorticoid receptor contribute to muscular atrophy (18, 19). In the present study, the administration of DEX led to a decrease of muscle mass, which verified the Dex-induced muscle atrophy model.

Stem cells with multi-differentiation potential are considered as an effective treatment for many kinds of diseases. Although MSCs transplantation increased the regeneration of skeletal muscle, the cells did not differentiate into skeletal myofibers indicating a paracrine role of MSCs in muscle regeneration (5). Accumulating evidence confirm that exosomes exert an essential role for cell-to-cell and organ-to-organ communication *via* endocrine signaling. It has been reported that stem cell-derived exosomes inhibited doxorubicin-induced muscle toxicity through decreasing pro-inflammatory cytokines. In addition, stem cell-derived exosomes showed a significant reduction in muscular atrophy and fibrosis in doxorubicin treated mice (20). MSC-derived exosomes have been reported to increase the expression of MyoG and MyoD1 of C2C12 cells, indicating that MSC-derived exosomes had the effect to promote myogenesis (12). In the present research, the diameter of C2C12 myotubes decreased after DEX treatment, which was partly rescued by BMSC-Exos administration. Furthermore, BMSC-Exos inhibited DEX-induced skeletal muscle atrophy in mice. BMSCs-Exos administration significantly ameliorated the up-regulation of atrogin-1 and MuRF1 gene level and the down-regulation of MyoG and MyoD gene level. Atrogin-1 and MuRF1 are atrophy-related genes. The level of atrogin-1 and MuRF1 were up-regulated at the early stage of muscle wasting and keeping high level throughout the period of muscle atrophy. Atrogin-1 and MuRF1 knock-out showed a protective role of denervation-induced muscle atrophy in mice (21, 22). Atrogin-1 and MuRF1 are regulated by Forkhead box O 1(FoxO1) during muscle atrophy (23, 24). FoxO1 is a member of Forkhead box O (FoxO) proteins, which are transcriptional factors that play critical roles in the regulation of skeletal muscle mass and myogenic differentiation (25–27). In our study, the level of Foxo-1 was down regulated by BMSC-Exos indicating the protective effect of BMSC-Exos was mediated by FoxO1.

Recently, miRNAs in exosomes have been considered as a major transport mechanism during the process of intercellular communication (28). MicroRNAs are short, non-coding RNAs that are involved in cellular processes due to their ability to inhibit the function of target mRNAs at the post-transcriptional level instead of translating into proteins. In recent decades, miRNAs have been confirmed to play vital roles in the development of sarcopenia. Clinical evidence showed that the expression of 13 miRNAs was changed in the muscles or blood of sarcopenia patients (1). Meanwhile, 10 miRNAs were changed in muscles of rodent models of sarcopenia (1). These miRNAs were associated with the expression levels of many signaling molecules, including FoxO family, sirtuin 1 and insulin-like growth factor-1 (29). MiR-486 is a muscle-enriched miRNA and over-expression of miR-486 improves the physiological function of the muscle and its strength (30). A previous study found that miR-486-5p was significantly decreased in both aged senescence accelerated mouse-prone 8 (SAMP8) mice and late passage C2C12 cells (31). Previous studies proved that miR-486-5p is highly expressed in exosomes derived from BMSCs (32). Moreover, overexpression of miR-486 inhibit PTEN, accounting for increased AKT and diminished FOXO1, inhibited proteolysis and resulted in muscle growth (33). In the present study, BMSC-Exos treatment increased the expression of miR-486-5p in C2C12 cells. In order to further verify the role of BMSC-Exos-associated miR-486-5P in Dex-induced muscle atrophy, miR-486-5p inhibitor was further used to reduce the level of miR-486-5p in BMSC-Exos. The results showed that the expression of FoxO1 was higher in the miR-486 inhibited-BMSC-Exos treated C2C12 cells when compared with inhibitor control Exos group. At the same time, the results showed that the protective role of BMSC-Exos against Dex-induced muscle atrophy was partially eliminated by miR-486-5p inhibitor. In animal experiments, the muscle cross-sectional area of miR-486 inhibited-BMSC-Exos treated was less than that of inhibitor control group. Meanwhile, BMSC-Exos increases myotube diameter compared with control group. This may relate to the increased differentiation and fusion of myoblasts after the treatment of BMSC-Exos. FOXO1 also plays an important role in myogenesis through regulating MyoG and MyoD expression. Taken together, the results of our study indicating that exosomes isolated from the BMSCs could attenuates dexamethasone-induced muscle atrophy through miR-486-5p/FoxO1.

In summary, we found that BMSC-Exos effectively inhibited DEX-induced myotube atrophy and muscle wasting both *in vitro* and *in vivo*. Further exploration of underline mechanism proved that the BMSC-Exos delivered miR-486-5p to muscle cells, which reduced the expression of FoxO1 in nucleus, and reduced the expression of atrophy related genes. Our exploration on the functional mechanism of BMSC-Exos is of much significance to developing a new biotherapy of muscle atrophy based on exosomes. This research preliminarily explored a new approach to ameliorate muscle atrophy which need further exploration.

## Data Availability Statement

The raw data supporting the conclusions of this article will be made available by the authors, without undue reservation.

## Ethics Statement

The animal study was reviewed and approved by the ethics committee of the Third Hospital of Hebei Medical University.

## Author Contributions

PX and SJL designed the study. ZL, SLL, and CL did the experiments and collected the data. TL, YL, NW, and XB carried out the data analysis. ZL drafted the manuscript. All authors contributed to the article and approved the submitted version.

## Funding

This study was supported by Basic Research Program for Beijing-Tianjin-Hebei Coordination (No. 19JCZDJC65500[Z]), Osteoporosis Program for Young Doctors (No. GX20191107), Medical Application Technology Program of Hebei Province (No. G2019008), Tianjin Outstanding Youth Fund Project (No. 20JCJQIC00230) and Sichuan Science and Technology Program (No. 2021YFH0004).

## Conflict of Interest

The authors declare that the research was conducted in the absence of any commercial or financial relationships that could be construed as a potential conflict of interest.

## Publisher’s Note

All claims expressed in this article are solely those of the authors and do not necessarily represent those of their affiliated organizations, or those of the publisher, the editors and the reviewers. Any product that may be evaluated in this article, or claim that may be made by its manufacturer, is not guaranteed or endorsed by the publisher.
